# The complete mitochondrial genome sequences of five Otophysi species (Vertebrata, Teleostei)

**DOI:** 10.1080/23802359.2019.1693294

**Published:** 2019-11-21

**Authors:** Rodrigo Milan Calegari, Pedro Henrique Mira Rodrigues, Rodrigo Zeni dos Santos, Fausto Foresti, Ricardo Utsunomia, Fábio Porto-Foresti

**Affiliations:** a Departamento de Ciências Biológicas, Faculdade de Ciências, Universidade Estadual Paulista, Bauru, Sao Paulo, Brazil;; b Departamento de Morfologia, Instituto de Biociências, Universidade Estadual Paulista, Botucatu, Sao Paulo, Brazil;; c Departamento de Genética, Universidade Federal Rural do Rio de Janeiro, Seropedica, Brazil

**Keywords:** Characiformes, Gymnotiformes, neotropical fish

## Abstract

Complete mitochondrial genomes of the characiform fishes *Astyanax fasciatus*, *Astyanax altiparanae*, *Hoplias malabaricus* (Karyomorph A) and the Gymnotiformes species *Gymnotus sylvius* and *Gymnotus cuia* were characterized in the present study. The whole mitogenomes varied from 16,400bp (*A. fasciatus)* to 17,730 bp (*A. altiparanae*) long and all of them consisted of 13 protein-coding genes, 22 tRNAs, 2 rRNAs genes, a control region, and origin of light-strand replication. The gene order was similar among all the analyzed species. The nucleotide content of all mitogenomes was also similar, with 29.58–30.95% for A, 27.02–28.65% for T, 26.29–29.99% for C, and 14.41–15.67% for G.

Otophysi is a major freshwater fish clade that exhibits a remarkable species diversity, counting over 10,000 species, that comprises the orders Cypriniformes, Gymnotiformes, Siluriformes and Characiformes (Betancur et al. 2018). In the late decades, the monophyly of groups within Otophysi was intensively tested using distinct molecular phylogenetics approaches, from single gene to large multilocus datasets (Oliveira et al. 2011; Betancur et al. 2018). Here, we provided the complete mitogenomes of *A. fasciatus*, *A. altiparanae*, *H. malabaricus* (karyomorph A), *G. sylvius* and *G. cuia*.

Species were collected at different sites and deposited at the fish collection of the Laboratório de Biologia e Genética de Peixes, Botucatu, São Paulo, Brazil (LBP) or at the Laboratório de Genética de Peixes, Bauru, São Paulo, Brazil (LAGENPE), *H. malabaricus* (LAGENPE11105), *A. altiparanae* (LAGENPE11102) and *G. cuia* (LBP38772) at Batalha river (Tietê river basin, 22°23′39″S, 49°06′35″W), *G. sylvius* (LBP33925) at Veu da Noiva waterfall (Paranapanema river basin, 22°59′23″S, 48°25′31″W) and *A. fasciatus* (LBP75313) at Córrego das Araras stream (Mogi-Guaçu river basin, 22°27′6″S, 49°14′25″W). Total genomic DNA from liver was extracted using the Wizard Genomic DNA Purification Kit (Promega (Madison, Wi, USA)). Whole genome sequencing was performed with Illumina platforms (MiSeq or Hiseq2500). The mitogenomes were assembled using NOVOPlasty (Dierckxsens et al. 2017) and the annotations were carried out using MitoAnnotator (Iwasaki et al. 2013). The lengths of the complete mitochondrial genomes varied from 16,400 (*A. fasciatus*) to 17,730 (*A. altiparanae*) and all of them contained 22 transfer RNA genes (tRNAs), 13 protein-coding genes (PCGs), two ribosomal genes, and a control region. The gene arrangements of the presented mitogenomes are similar among each other and to the typical arrangement of vertebrates. Most of the genes were encoded on the light-strand, except for eight tRNA genes and *ND6*, which are encoded in the heavy-strand. All PCGs had ATG as initiation codon, except for COI and the ATPase 6 gene of both *Astyanax* species, which used GTG. The annotated sequence files were submitted to NCBI (Accession No. MN583176–MN583180).

In order to illustrate the phylogenetic position of the five newly sequenced mitogenomes, their phylogenetic relationships were estimated using a concatenated dataset of 13 protein-coding genes from several Otophysi species. The 13 protein-coding genes were extracted and aligned with the MUSCLE algorithm (Edgar 2004), and then, trees were calculated using the maximum likelihood method in PhyML (Guindon et al. 2010) with a GTR + I +G model and 1000 bootstrap replicates. In the phylogenetic analyses, ancient nodes were weakly supported, as expected since the phylogeny of Otophysi is highly controversial (Betancur et al. 2018), but recent nodes were strongly supported, as evidenced in Figure 1.

**Figure 1.**
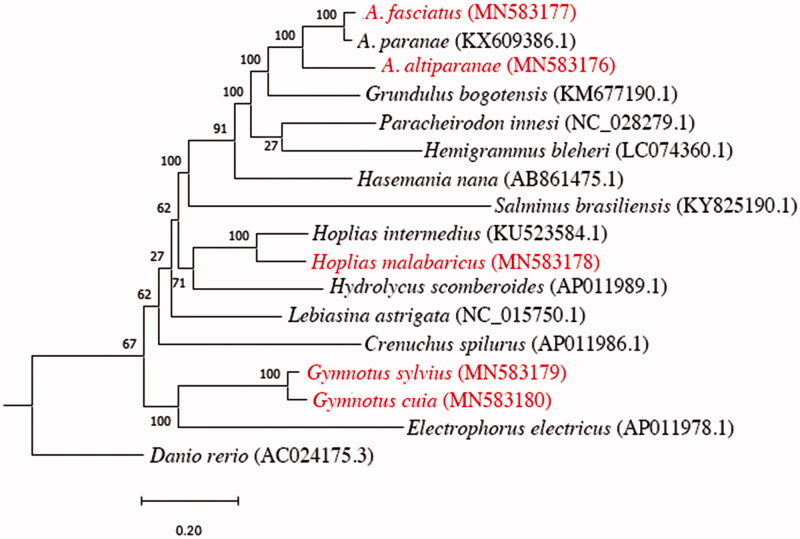
Maximum likelihood tree showing the phylogenetic relationships between the assembled mitogenomes (species in red) and other available mitogenomes for Characiformes species, using as external group the zebrafish.
